# Development and Validation of a Bedside Risk Assessment for Sustained Prescription Opioid Use After Surgery

**DOI:** 10.1001/jamanetworkopen.2019.6673

**Published:** 2019-07-10

**Authors:** Muhammad Ali Chaudhary, Nizar Bhulani, Elzerie C. de Jager, Stuart Lipsitz, Nicollette K. Kwon, Daniel J. Sturgeon, Quoc-Dien Trinh, Tracey Koehlmoos, Adil H. Haider, Andrew J. Schoenfeld

**Affiliations:** 1 Center for Surgery and Public Health, Department of Surgery, Brigham and Women’s Hospital, Harvard Medical School, Boston, Massachusetts; 2 Center for Surgery and Public Health, Division of Urology, Department of Surgery, Brigham and Women’s Hospital, Harvard Medical School, Boston, Massachusetts; 3 Department of Preventive Medicine & Biostatistics, Uniformed Services University of the Health Sciences, Bethesda, Maryland; 4 Center for Surgery and Public Health, Department of Orthopaedic Surgery, Brigham and Women’s Hospital, Harvard Medical School, Boston, Massachusetts

## Abstract

**Importance:**

The increased use of prescription opioid medications has contributed to an epidemic of sustained opioid use, misuse, and addiction. Adults of working age are thought to be at greatest risk for prescription opioid dependence.

**Objective:**

To develop a risk score (the Stopping Opioids After Surgery score) for sustained prescription opioid use after surgery in a working-age population using readily available clinical information.

**Design, Setting, and Participants:**

In this case-control study, claims from TRICARE (the insurance program of the US Department of Defense) for working-age adult (age 18-64 years) patients undergoing 1 of 10 common surgical procedures from October 1, 2005, to September 30, 2014, were queried. A logistic regression model was used to identify variables associated with sustained prescription opioid use. The point estimate for each variable in the risk score was determined by its β coefficient in the model. The risk score for each patient represented the summed point totals, ranging from 0 to 100, with a lower score indicating lower risk of sustained prescription opioid use. Data were analyzed from September 25, 2018, to February 5, 2019.

**Exposures:**

Exposures were age; race; sex; marital status; socioeconomic status; discharge disposition; procedure intensity; length of stay; intensive care unit admission; comorbid diabetes, liver disease, renal disease, malignancy, depression, or anxiety; and prior opioid use status.

**Main Outcomes and Measures:**

The primary outcome was sustained prescription opioid use, defined as uninterrupted use for 6 months following surgery. A risk score for each patient was calculated and then used as a predictor of sustained opioid use after surgical intervention. The area under the curve and the Brier score were used to determine the accuracy of the scoring system and the Hosmer-Lemeshow goodness-of-fit test was used to evaluate model calibration.

**Results:**

Of 86 356 patients in the analysis (48 827 [56.5%] male; mean [SD] age, 46.5 [14.5] years), 6365 (7.4%) met criteria for sustained prescription opioid use. The sample used for model generation consisted of 64 767 patients, while the validation sample had 21 589 patients. Prior opioid exposure was the factor most strongly associated with sustained opioid use (odds ratio, 13.00; 95% CI, 11.87-14.23). The group with the lowest scores (<31) had a mean (SD) 4.1% (2.5%) risk of sustained opioid use; those with intermediate scores (31-50) had a mean (SD) risk of 14.9% (6.3%); and those with the highest scores (>50) had a mean (SD) risk of 35.8% (3.6%).

**Conclusions and Relevance:**

This study developed an intuitive and accessible opioid risk assessment applicable to the care of working-age patients following surgery. This tool is scalable to clinical practice and can potentially be incorporated into electronic medical record platforms to enable automated calculation and clinical alerts that are generated in real time.

## Key Points

**Question:**

Can a risk score for sustained prescription opioid use after surgery be developed for a working-age population using readily available clinical information?

**Findings:**

In this case-control study of 86 356 patients undergoing 1 of 10 common surgical procedures, prior opioid exposure was the factor most strongly associated with sustained opioid use. The group with the lowest Stopping Opioids After Surgery scores (<31) had a mean 4.1% risk of sustained opioid use; the group with intermediate scores (31-50) had a mean risk of 14.9%; and the group with the highest scores (>50) had a mean risk of 35.8%.

**Meaning:**

The scoring system developed in this study may inform the risk of sustained prescription opioid use after surgery and be scalable to clinical practice.

## Teaser

This case-control study describes the development and validation of a risk score, the Stopping Opioids After Surgery Score, for sustained prescription opioid use after surgery in a population of patients participating in a US Department of Defense insurance program.

## Introduction

Since the year 2000, the United Sates has experienced an epidemic of prescription opioid use, abuse, and dependence.^1,2,3^ With more than 259 million opioid prescriptions issued in 2012, the use of prescription opioid pain medications is now 4 times higher than it was in 1999.^4,5^ While representing less than 5% of the global population, the US population is thought to be responsible for more than 80% of opioid consumption worldwide.^6^ Continued prescription opioid use has been implicated in higher rates of drug poisoning,^3^ with an associated cost of more than $53 billion in the form of health care expenditures, addiction treatment, criminal justice costs, and lost productivity.^7,8^

Surgical episodes are known to be associated with high incidence of acute pain and prescription opioid use.^3,7,8,9,10,11^ Prior research has found that postsurgical opioid prescriptions are issued in as many as 99% of cases and surgeons are among the most common prescribers of opioids.^3,9,10,11,12^ If prescription opioid use is extended beyond 12 weeks, the addiction rate may be as high as 50%.^13^ Although several studies have worked to identify factors associated with sustained prescription opioid use in the surgical population, their direct impact on clinical practice is limited.^8,9,10,11,12,13,14,15^ This is because many of the prognostic factors identified are not easily accessible at the point of care such that a clinician can rapidly apply them to decision-making. Furthermore, estimates in smaller samples may be influenced by the prevalence of sustained prescription opioid use within that population, as well as variation in the pretest probability of the outcome. A practical, easy way to calculate the risk of sustained opioid use in patients undergoing surgery is not presently available, to our knowledge. While developing interventions capable of mitigating long-term use remains a priority, we believe that the efficacy of these efforts is predicated on identifying the individuals most likely to become sustained prescription users following surgical interventions.

In this context, we sought to develop a robust risk score, the Stopping Opioids After Surgery (SOS) score, for sustained prescription opioid use after surgery using readily accessible clinical information that could be directly applied to decision-making and planning following discharge.

## Methods

### Data Source

We used TRICARE claims data (October 1, 2005, to September 30, 2014) from the Military Health System Data Repository. TRICARE is the health insurance program of the US the Department of Defense and covers more than 9.5 million active-duty and retired military personnel and their dependent beneficiaries.^12,16^ Care is administered either through civilian medical facilities or health care entities maintained by the Department of Defense.^16,17^ The mechanisms through which TRICARE claims are collected, stored, and accessed has been described in prior literature.^11,12,15,16,18,19^ It is important to note that TRICARE is not responsible for care administered through the Veterans Health Administration or care provided in combat zones.^16,17^ TRICARE data have been successfully used in the past to evaluate opioid use, health care disparities, and surgical care quality.^11,12,15,19^ The demographic characteristics of the covered population broadly approximates that of US adults younger than 65 years.^16,17^ The study protocol was deemed exempt by the institutional review board of the Uniformed Services University of the Health Sciences and the Partners institutional review board. All data were deidentified before analysis. Analysis was conducted from September 25, 2018, to February 5, 2019. The study followed Strengthening the Reporting of Observational Studies in Epidemiology (STROBE) reporting guideline.

### Study Population

We queried TRICARE claims of working-age adult (age 18-64 years) patients undergoing 1 of 10 common surgical procedures representing the disciplines of general surgery (appendectomy, inguinal herniorrhaphy, and colectomy), cardiovascular surgery (coronary artery bypass grafting), urology (transurethral resection of prostate, nephrectomy, and radical cystectomy), and orthopedics (total knee arthroplasty, total hip arthroplasty, and hip fracture repair) using *International Classification of Disease, Ninth Revision* (*ICD-9*) procedure codes. Procedures were selected owing to their high frequency and the fact that they have been considered representative of the respective surgical subspecialties in prior work.^19^ Procedures were classified as minor (appendectomy, inguinal herniorrhaphy, and transurethral resection of prostate) and major (colectomy, coronary artery bypass grafting, nephrectomy, radical cystectomy, total knee arthroplasty, total hip arthroplasty, and hip fracture repair) based on complexity and degree of surgical invasiveness.^15,19^ Major surgical procedures are those that require access to major organ spaces or the resection of osseous structures. To ensure 6 months of preoperative and postoperative opioid surveillance, patients who underwent a surgical procedure in the first 6 months of 2006 and the last 6 months of 2014 were excluded. In addition, patients who died during hospitalization and those who were eligible for Medicare were also excluded.^16^

### Variable Definitions

We surveyed all claims data for patients who met inclusion criteria and recorded age at the time of surgery, race (classified as white, black, or other), biological sex, marital status, sponsor rank (an established proxy for socioeconomic status in TRICARE data with enlisted sponsor rank considered indicative of lower socioeconomic strata^16,19,20^), discharge disposition (home or nonhome), procedure type (major or minor), length of hospitalization, intensive care unit admission, and preoperative diagnoses (defined by *ICD-9* code) of diabetes, liver disease, renal disease, malignancy, depression, and anxiety. Length of hospitalization was dichotomized at 3 days based on the median length of hospitalization in the cohort.

### Surveillance for Prescription Opioid Use

Information on prescriptions billed to TRICARE is available through the Military Health System Data Repository.^15,16,18^ In line with prior studies,^11,12,15,18^ we used the Drug Enforcement Administration’s list of Schedule II (high abuse potential) and III (moderate risk of dependence) opioid combinations to query prescription data for patients starting 6 months prior to the date of surgery and extending to 6 months following that date. The number of tablets issued and length of the prescription, assuming medications were taken as ordered, were recorded. Based on prior definitions,^11,12,18^ the criteria for sustained prescription opioid use in this study consisted of 6 months of continuous prescription opioids billed to TRICARE without an interruption exceeding 7 days.

### Statistical Analysis

The primary outcome in this analysis was sustained prescription opioid use according to the stated criteria. All clinical and demographic covariates were considered eligible for inclusion in the model. As extent of opioid use is notoriously difficult to quantify,^3^ we considered any opioid use in the 6 months prior to the surgery as a positive finding, irrespective of the type of opioid prescribed or the duration of preoperative exposure. Study variables were summarized by frequencies and percentages. The study cohort was randomly divided into a 75% sample for model generation and a 25% sample for validation (Figure).

**Figure.**
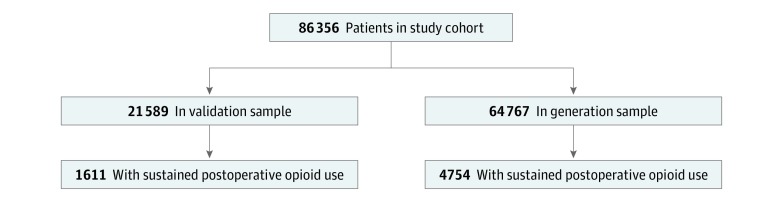
Schematic of Sample Selection for Model Generation and Risk-Score Validation

A logistic regression model was used to identify variables associated with sustained prescription opioid use in the 75% sample. The large sample size of our cohort allowed us to include all a priori study variables in the logistic regression model. Variable elimination was performed for factors that did not achieve statistical significance in the multivariable test as defined by 2-sided *P* < .05. Area under the receiver operator characteristic curve (AUC) was used to evaluate model performance. The covariates were coded such that all β coefficients were positive. Then the points that each variable contributed to the risk score was determined by comparing the β coefficient of the variable to the overall sum of coefficients in the model, multiplying by 100, and rounding to the nearest integer to facilitate calculation. The risk score calculated for each patient represented the summed point totals from all variables present, with a range of 0 to 100 (lower score indicates lower risk of sustained prescription opioid use).

The risk score was internally validated using the remaining 25% sample previously held out. A risk score for each patient was calculated and then used as a predictor of sustained opioid use after surgical intervention. The AUC was evaluated to determine whether there was any change in model performance. Additionally, the Brier score was calculated to determine the accuracy of the scoring system and the Hosmer-Lemeshow goodness-of-fit test was used to evaluate model calibration across both samples. All analyses were performed using STATA statistical software version 14.0 (StataCorp).

## Results

We included 86 356 patients in this analysis (48 827 [56.5%] male; mean [SD] age, 46.5 [14.5] years), with 6365 (7.4%) meeting our criteria for sustained prescription opioid use after surgery. The sample used for model generation consisted of 64 767 patients, while the validation sample had 21 589 patients. Both cohorts possessed relatively equal proportions of patients meeting the study definition of sustained prescription opioid use after surgery (Figure).

### Risk Score Development

Numerous clinical and sociodemographic characteristics were associated with sustained prescription opioid use (Table 1), with preoperative opioid use having the strongest association. The final multivariable model included patient age, biological sex, sponsor rank (our proxy for socioeconomic status), discharge status, procedure type, length of stay, depression and/or anxiety, and preoperative opioid use (Table 2). Sustained opioid use within 6 months preceding the surgical intervention was the factor most strongly associated with postsurgical sustained opioid use (odds ratio, 13.00; 95% CI, 11.87-14.23) and received the highest individual risk score (36 points). This was followed by prior opioid exposure within 6 months preceding the surgery (odds ratio, 3.21; 95% CI, 2.96-3.47) and nonhome discharge (odds ratio, 2.14; 95% CI, 1.62-2.83) with scores of 17 and 11 points, respectively.

**Table 1.** Patient Sociodemographic and Clinical Characteristics

Characteristic	No. (%)			*P* Value
	Total Cohort (N = 86 356)	Sustained Opioid Use		
		Yes (n = 6365)	No (n = 79 991)	
Age, y				
18-24	10 628 (12.3)	379 (6.0)	10 249 (12.8)	<.001
25-34	11 834 (13.7)	599 (9.4)	11 235 (14.0)	
35-44	9990 (11.6)	709 (11.1)	9281 (11.6)	
45-54	18 609 (21.5)	1570 (24.7)	17 039 (21.3)	
55-64	35 295 (40.9)	3108 (48.8)	32 187 (40.2)	
Race				
White	49 338 (57.1)	3417 (53.7)	45 921 (57.4)	<.001
Black	9713 (11.2)	630 (9.9)	9083 (11.4)	
Others	10 458 (12.1)	648 (10.2)	9810 (12.3)	
Missing	16 847 (19.5)	1670 (26.2)	15 177 (19.0)	
Female	37 529 (43.5)	3483 (54.7)	34 046 (42.6)	<.001
Married	70 113 (81.2)	5495 (86.3)	64 618 (80.8)	<.001
Lower socioeconomic status	67 552 (78.2)	5319 (83.6)	62 233 (77.8)	<.001
Nonhome discharge	700 (0.8)	89 (1.4)	611 (0.8)	<.001
Procedure category				
Minor	32 248 (37.3)	1359 (21.4)	30 889 (38.6)	<.001
Major	54 108 (62.7)	5006 (78.6)	49 102 (61.4)	
Length of stay, d				
≤3	55 462 (64.2)	3724 (58.5)	51 738 (64.7)	<.001
>3	30 894 (35.8)	2641 (41.5)	28 253 (35.3)	
Intensive care unit admission	6290 (7.3)	514 (8.1)	5776 (7.2)	.01
Diabetes	10 032 (11.6)	960 (15.1)	9072 (11.3)	<.001
Liver disease	467 (0.5)	42 (0.7)	425 (0.5)	.18
Renal disease	1274 (1.5)	103 (1.6)	1171 (1.5)	.33
Any malignant neoplasm	6468 (7.5)	554 (8.7)	5914 (7.4)	<.001
Depression	5372 (6.2)	759 (11.9)	4613 (5.8)	<.001
Anxiety	2512 (2.9)	373 (5.9)	2139 (2.7)	<.001
Prior opioid use				
No use	47 382 (54.9)	46 095 (57.6)	1287 (20.2)	<.001
Prior opioid exposure	32 309 (37.41)	29 262 (36.6)	3047 (47.9)	
Prior sustained opioid use	6665 (7.72)	4634 (5.8)	2031 (31.9)	

**Table 2.** Multivariable Model With Associated Risk Score for Each Included Variable^a^

Characteristic	Adjusted Odds Ratio (95% CI)	Score
Age, y		
18-24	1 [Reference]	0
25-34	1.21 (1.03-1.43)	3
35-44	1.37 (1.16-1.61)	4
45-54	1.33 (1.13-1.55)	4
55-64	1.33 (1.14-1.55)	4
Sex		
Male	1 [Reference]	0
Female	1.22 (1.14-1.30)	3
Discharge status		
Home	1 [Reference]	0
Nonhome	2.14 (1.62-2.83)	11
Socioeconomic status		
High	1 [Reference]	0
Low	1.43 (1.31-1.55)	5
Procedure category		
Minor	1 [Reference]	0
Major	1.29 (1.18-1.42)	4
Length of stay, d		
≤3	1 [Reference]	0
>3	1.08 (1.01-1.16)	1
Depression	1.35 (1.22-1.50)	4
Anxiety	1.35 (1.17-1.56)	4
Prior opioid use		
No use	1 [Reference]	0
Prior opioid exposure	3.21 (2.96-3.47)	17
Prior sustained opioid use	13.00 (11.88-14.23)	36
Total score		100

^a^ The multivariable logistic regression model was adjusted for variables selected through backward stepwise regression and risk scores for each variable were calculated using the log odds of the variable divided by the sum of the log odds of the model, multiplied by 100 and rounded to the nearest integer.

The opioid risk score was further stratified into 3 categories (low, intermediate, and high) based on the distribution of the risk scores and the incidence of sustained opioid use within each group (Table 3). The low-risk cohort (score <31) had a mean (SD) 4.1% (2.5%) predicted risk of sustained prescription opioid use, the intermediate group (score 31-50) had a mean (SD) risk of 14.9% (6.3%), and the high-risk category (score >50) had a mean (SD) risk of 35.8% (3.6%) for sustained use after surgery.

**Table 3.** Risk Score Stratification Into Risk Categories

Opioid Risk Score, Range	Risk Category	Likelihood of Sustained Opioid Use, Mean (SD), %
<31	Low	4.1 (2.5)
31-50	Intermediate	14.9 (6.3)
>50	High	35.8 (3.6)

### Risk Score Validation

In the validation sample, the Brier score for the model was 0.08, indicative of good performance. There was no change in the risk score’s discriminative capacity between the sample used to generate the tool and the cohort used for validation, with both demonstrating an AUC of 0.76 (eFigure in the Supplement). There was no statistically significant evidence of lack of fit in the samples used for model generation (*P* = .58) or validation (*P* = .96).

## Discussion

Since The Joint Commission introduced its mandatory pain evaluation guidelines in 2001, the use of prescription opioid medications has increased exponentially and contributed to an epidemic of sustained opioid use, abuse, misuse, and addiction.^3,7,8,9,11,12,18,21,22,23,24,25,26^ Prior research regarding opioid use in patients undergoing surgery has generally only characterized factors associated with sustained use or focused on prescribing practices after particular procedures.^8,9,10,11,12,13,14,15,22,23,24,25^ Such work is not only limited by the prevalence of opioid use in the communities under study, but also influenced by the predictors considered and challenges to the application of such findings in everyday practice. The parallel influence of risk factors is notoriously difficult to parse and comorbidity scores, morphine milligram equivalents, and disease severity are challenging to calculate in real time during hospital encounters.^3^ The objective of this work was to develop an accessible and intuitive battery of clinical criteria that could rapidly be used to calculate the risk of prescription opioid use in patients following surgery. We have termed the resultant tool the SOS score.

We were able to include more than 86 000 surgical events using a data source composed of records of patients treated in disparate clinical contexts across the United States.^16,17^ As previously documented, the geographic, sociodemographic, educational, vocational, and occupational variation encountered in the population insured through TRICARE is representative of the US population younger than 65 years.^16,17^ The working-age population under study also encompasses cohorts maintained to be at greatest risk of prescription opioid misuse and dependence.^3^ These facts, coupled with our inclusion of prescription opioid data from a variety of commonly performed surgical procedures, support our position that the SOS score can be applied to clinical practice, irrespective of the hospital location, environment of care, or the procedure being performed. Although the pretest probability of sustained prescription opioid use cannot be empirically evaluated here, there is no evidence to suspect a fundamental difference in baseline risk between patients insured through TRICARE and the US general population.

Forty-five percent of the study population was exposed to opioids within the 6 months leading up to their surgical procedure, a figure comparable to other work regarding preoperative opioid use in large surgical cohorts.^14,22,23,25^ The prevalence of sustained prescription opioid use up to 6 months following surgery in our sample is aligned with outside estimates^11,12^ and also falls within the reported range of dependence following long-term use of prescription opioids (3%-45%).^3^ Furthermore, nearly all clinical variables included in the risk score have been independently substantiated as risk factors for prolonged prescription opioid use.^3,26^ For example, in a series of patients treated with elective spine surgery, Schoenfeld et al^11^ reported that patient age, duration of preoperative opioid use, and history of psychiatric disorders were associated with sustained prescription opioid use. Similar sociodemographic characteristics were identified by Chaudhary et al^12^ in patients treated for orthopedic traumatic injuries. These authors^12^ also determined that hospital length of stay was associated with increased risks of sustained use. Scully et al^15^ previously determined that high-intensity surgical procedures were associated with a longer duration of prescription opioid use, another aspect incorporated into the SOS score. The association of low socioeconomic status with the risk of sustained prescription opioid use has been highlighted in several investigations evaluating a broad spectrum of surgical interventions.^3,11,12^

We believe that we have achieved our stated objective of developing an accessible and pragmatic risk score for postsurgical opioid use that consists of readily available data points and can be applied to clinical practice. The objective of this effort was to create a risk stratification tool for better discharge planning and not to generate a precise epidemiological prediction model. Nearly all variables included in the score are easy to determine and convey to patients, with the possible exception of socioeconomic status. Our proxy for low socioeconomic status, junior enlisted sponsor rank, is well substantiated in work relying on TRICARE data,^16,17,20^ but an exact corollary does not exist in the general population. Other accepted markers for socioeconomic status, such as insurance status or type of employment, could likely be used in the place of sponsor rank, but this remains to be tested. At present, the SOS score may be used to determine the probability of sustained use and, in high-risk scenarios, could suggest consideration of opioid-sparing strategies^3^ for postoperative pain management. The score could also be used as objective support for clinical decisions, such as limiting the amount of prescription opioids issued at discharge, that may otherwise be difficult to rationalize to patients. An automated score calculated by an algorithm that pulls characteristics directly from the electronic medical record is also envisioned as a means to immediately modulate opioid prescribing practices at the time of discharge. A pilot program along these lines has been described elsewhere^3^ but does not use a comprehensive risk assessment tool such as the SOS.

### Limitations

This study has limitations. We recognize that, as this study relied on administrative data, there is the potential for coding errors or inaccurate reporting of claims to affect results, and the risk of this bias cannot be quantified or addressed. Furthermore, we are limited to consider only prescription opioid use, with the assumption that medications were used as directed by the clinician.^11,12,15,18^ Our models cannot address misuse, diversion, or the use of illegal narcotics.^21^ Similarly, we are not able to reliably assess preoperative history of substance abuse or alcohol use disorder, factors that are known to be associated with the risk of prescription opioid dependence.^3,26^ These aspects of a patient’s history may be difficult to assess with accuracy and, thus, impede our goal of compiling an accessible risk score that is easily calculated at the bedside. For this same reason, we restricted our preoperative opioid exposure variable to any use in the 6 months prior to surgery, as opposed to stratifying by type of opioid, length of exposure, or morphine milligram equivalents. This effort was not designed to identify which prognostic factors should be screened when considering prescription opioid dependence, but rather was intended to develop an informative score easily calculated from universally accessible patient characteristics. The SOS score’s performance was unchanged between the samples used for generation and validation in this study, which is encouraging as the rate of sustained opioid use was identical in both cohorts. We recognize, however, that both samples were prepared from patients insured through TRICARE and the score’s utility in external populations needs to be assessed through additional research. At present, the tool is likely not applicable to patients not undergoing surgery or those over age 65 years, and its value in characterizing opioid use among individuals receiving interventions substantially different from those considered here remains to be determined.

## Conclusions

We have developed an intuitive and accessible opioid risk score that is applicable to the care of patients following surgery. The SOS score can identify patients at low, intermediate, and high risk for sustained prescription opioid use after surgery. This tool is scalable to clinical practice and can potentially be incorporated into electronic medical record platforms to enable automated calculation and clinical alerts that are generated in real time. This study can be used as a general literature citation supporting the use of the SOS in future investigations.

## Notes

Supplement. **eFigure.** Area Under the Receiver Operator Curve for Generation and Validation Samples
