# Supplementary material for: Development and Validation of a Bedside Risk Assessment for Sustained Prescription Opioid Use After Surgery
Source: JAMA Netw Open. 2019 Jul 10;2(7):e196673. doi: 10.1001/jamanetworkopen.2019.6673 (PMC6624809; doi:10.1001/jamanetworkopen.2019.6673)
Supplement: Supplement. — eFigure. Area Under the Receiver Operator Curve for Generation and Validation Samples [file jamanetwopen-2-e196673-s001.pdf]

Supplementary Online Content

Chaudhary MA, Bhulani N, de Jager EC, et al. Development and validation of a bedside risk assessment for sustained prescription opioid use after surgery. *JAMA Netw Open*. 2019;2(7):e196673. doi:10.1001/jamanetworkopen.2019.6673

eFigure. Area Under the Receiver Operator Curve for Generation and Validation Samples

This supplementary material has been provided by the authors to give readers additional information about their work.

© 2019 Chaudhary MA et al. *JAMA Network Open.*

eFigure: Area Under the Receiver Operator Curve for Generation and Validation Samples

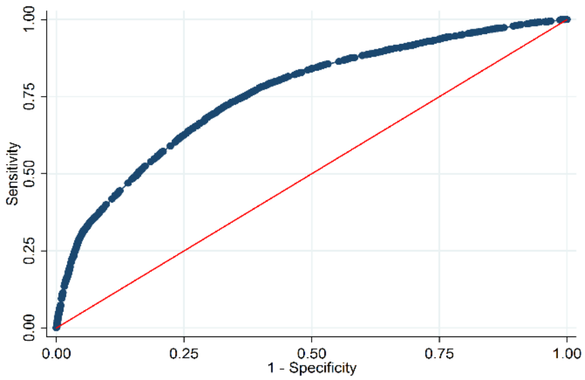

Area Under the Receiver Operator Characteristic (AUC) Curve for Model Generation Sample. AUC=0.76

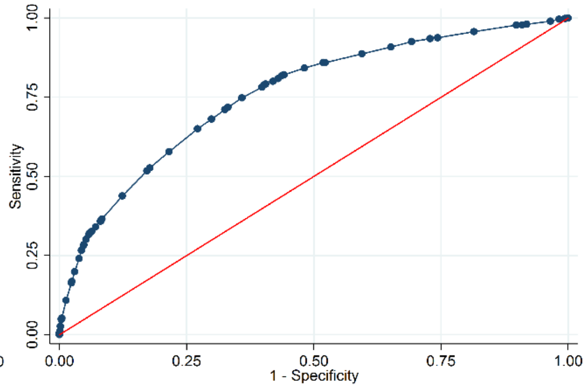

Area Under the Receiver Operator Characteristic (AUC) Curve for Risk Score Validation Sample. AUC=0.76

© 2019 Chaudhary MA et al. *JAMA Network Open.*
